# Development of a Novel Nomogram for Predicting Placenta Accreta in Patients With Scarred Uterus: A Retrospective Cohort Study

**DOI:** 10.3389/fmed.2019.00289

**Published:** 2019-12-17

**Authors:** Tian Yang, Na Li, Chong Qiao, Caixia Liu

**Affiliations:** ^1^Department of Obstetrics and Gynecology, Shengjing Hospital of China Medical University, Shenyang, China; ^2^Key Laboratory of Maternal-Fetal Medicine of Liaoning Province, Benxi, China; ^3^Key Laboratory of Obstetrics and Gynecology of Higher Education of Liaoning Province, Benxi, China

**Keywords:** placenta accreta, nomogram, risk factors, scarred uterus, China

## Abstract

**Objective:** The aim of this study was to develop a nomogram to predict the risk of placenta accreta in scarred uterus patients in China.

**Methods:** We retrospectively analyzed 8,371 singleton pregnancies with scarred uterus at Shengjing Hospital, affiliated with China Medical University. Two thirds of the patients were randomly assigned to the training set (*n* = 5,581), and one third were assigned to the validation set (*n* = 2,790). Multivariate logistic regression was performed by using the training set, and the nomogram was developed. Discrimination and calibration were performed by using both the training and validation sets.

**Results:** The multivariate logistic regression model identified number of previous cesarean section, number of vaginal bleeding, medication during pregnancy, and placenta previa as covariates associated with placenta accreta. A nomogram was developed to predict the risk of placenta accreta in the training set with a Harrell's C-index of 0.93 and 0.927 in the training set and validation set, respectively. Calibration of the nomogram predicted placenta accreta corresponding closely with the actual placenta accreta.

**Conclusion:** We developed a nomogram predicting the risk of placenta accreta in scarred uterus patients in China. Validation using both the training set and the validation set demonstrated good discrimination and calibration, suggesting good clinical utility.

## Introduction

With the introduction of the two-child policy in China, there have been increases in numbers of women with scarred uterus, who are therefore prone to uterine rupture, postpartum hemorrhage, placenta previa, and placenta accreta (PA) (1–5).

Placenta accreta refers to abnormal adherence of placental chorionic villi to the underlying myometrium with an absence of decidua basalis. It is classified into three types based on histopathology: PA when the chorionic villi just adhere to the myometrium, placenta increta when the chorionic villi invade the myometrium, and placenta percreta when the villi invade the full thickness of the myometrium and may even penetrate the uterine serosa (6–8). PA is associated with an increased risk of maternal morbidity and mortality, preterm birth, low birth weight, perinatal mortality, recurrent PA, uterine rupture, and postpartum hemorrhage in subsequent pregnancies (9–12). Considering this, it is important to establish a risk assessment tool for use during the earlier trimester in order to improve perinatal and maternal outcomes.

The incidence of PA has increased over the past 40 years along with the increased incidence of cesarean section (CS) (13–16), and is now reported to occur in 2–90 per 10,000 births (9, 13, 14, 17). Differences in study population and diagnostic criteria may account for this wide range. Another major risk factor for PA after CS is placenta previa (10, 12, 13, 16–20). Several other risk factors have also been reported, including cryopreserved embryo transfer, older maternal age, prior uterine surgery, parity, a higher body mass index, tobacco use, coexisting hypertension or diabetes, elevated second-trimester levels of α-fetoprotein and β-human chorionic gonadotropin, and a previous retained placenta or PA (16, 18–21).

Although many risk factors for PA in women with scarred uterus have been identified, to date, there has not been an established method for determining the effects of multiple risk factors and for determining the probability of PA in an individual woman in China, especially in northeast China. The purpose of this study was to combine the risk factors associated with PA into a prediction nomogram based on the data from a single large-volume institution.

## Methods

### Study Population

We undertook a retrospective study of women with scarred uterus who were patients at Shengjing Hospital, China Medical University, from January 2013 to December 2017. The clinical data were evaluated, and the inclusion criteria were as follows: scarred uterus (with at least one history of previous CS or history of myomectomy), singleton pregnancies, and termination of pregnancy at 24–44 weeks via vaginal delivery or CS. The exclusion criteria were as follows: cases with twins or multiple pregnancies, and termination of pregnancy at <24 weeks or more than 44 weeks.

The diagnosis of PA was made on clinical grounds, based on the objective difficulties experienced by the attending physician in removing the placenta during CS, or retained placenta in difficult and incomplete manual removal despite active management in the third stage of labor during vaginal delivery. If there was difficulty in diagnosis, pathological examination of remaining placenta attached to the uterus following manual removal was performed. The definition of PA included all pregnancies with partially or totally adherent placenta. There were only 102 cases further diagnosed by pathological examination.

This study was approved by the Ethical Committee of Shengjing Hospital, China Medical University.

### Investigated Clinical Characteristics

The clinical data came from the HIS system in our hospital and included basic patient demographics (age and body mass index), history of gestation (gravity, parity, number of previous vaginal delivery, number of previous CS, number of previous induction, and number of previous medical abortion), history of current illness, past medical history, and history of other obstetric diseases (including placenta previa, gestational diabetes, pregnancy-induced hypertension syndrome, etc.). To obtain information on any previous CS or myomectomy, we conducted a telephone follow-up of the patients (including time between last CS and pregnancy, whether previous CS was an elective CS, or whether previous CS was performed during labor).

Placenta previa was defined as the condition where the placenta, by lying in the lower uterine segment, partially or completely obstructs the internal orifice of the cervix.

Medications during pregnancy included antihypertensive drugs (labetalol, etc.), hypoglycemic drugs (insulin, etc.), hormones (progesterone, etc.), antibiotics, levothyroxine sodium, non-steroidal anti-inflammatory drugs (aspirin, etc.), and so on.

### Statistical Analysis

The analyses were performed using a split-sampling strategy: the overall sample was randomly split with stratification by center into training and validation sets. The former was used for model building, and the latter was used only for model testing purposes.

For model building, statistical analyses consisted of a series of steps. First, the categorical data were expressed as proportion and analyzed by chi-square test or Fisher's exact probability test. The continuous variables were expressed as mean ± SD and analyzed by two-sample *t*-test and the Mann–Whitney *U*-test if the data were not normally distributed. Second, multivariate logistic regression analyses were performed to assess features associated with PA. Variables tested included significant variables in the first step. Covariates were then removed by backward stepwise selection method until all covariates in the final model had a value of *P* < 0.05, and a binary logistic regression was developed. Finally, multivariate regression coefficients of the independent predictors of PA were then used to develop a nomogram predicting the probability of PA.

Nomogram validation consisted of discrimination (Harrell's C-index) and calibration (calibration plots) by both using the training set and the validation set. In general, a C-index value >0.75 was considered to represent relatively good discrimination. The validations for risk model were performed by bootstrapping 1,000 times in the training set and the validation set.

All statistical analyses were analyzed by the SPSS 22.0 software and R software version 3.5.1 (http://www.r-project.org) with the “sampling” and “rms” packages. We considered *P* < 0.05 as being statistically significant in a two-tailed test.

## Results

### Patient Characteristics

The study included 8,371 cases with scarred uterus, with the incidence of PA at 5.2%. Table 1 shows the basic characteristics of the training set (*N* = 5,581) and the validation set (*N* = 2,790). The mean ages were 33.28 ± 4.19 and 33.41 ± 4.18 years, and the times between last CS and pregnancy were 73.23 ± 45.25 and 75.01 ± 47.42 months, with 44.13% (2,463/5,581) and 43.30% (1,208/2,790) with missing data in the training set and validation set, respectively. In the training set and validation set, 90.84 and 90.25% of patients, respectively, had no bleeding during pregnancy. All in all, the patient's characteristics were similar between the training set and the validation set.

**Table 1:** Characteristics of the study population.

	**Training set (*N* = 5,581)**		**Validation set (*N* = 2,790)**		
**Variables**	**No. of patients**	**%**	**No. of patients**	**%**	***P-*value**
Age (years)	33.28 ± 4.19		33.41 ± 4.18		0.184
BMI before delivery	27.76 ± 3.69		27.73 ± 3.60		0.788
Gravity	3 (1–11)		3 (1–9)		0.354
Parity	1 (0–4)		1 (0–3)		0.954
Number of previous vaginal delivery	0 (0–4)		0 (0–2)		0.439
Number of previous cesarean section	1 (0–4)		1 (0–3)		0.959
Number of previous medical abortion	0 (0–8)		0 (0–3)		0.493
Number of previous artificial abortion	0 (0–7)		0 (0–6)		0.997
Number of previous induction	0 (0–4)		0 (0–4)		0.628
Myomectomy					0.229
Yes	328	5.88	146	5.23	
No	5,253	94.12	2,644	94.77	
History of gynecological surgery					0.319
Yes	335	6.00	183	6.56	
No	5,246	94.00	2,607	93.44	
Regular menstruation					0.862
Yes	5,137	92.04	2,565	91.94	
No	444	7.96	225	8.06	
Menstrual flow					0.586
Hypomenorrhea	174	3.12	80	2.87	
Normal	5,302	95.00	2,650	94.98	
Menorrhagia	105	1.88	60	2.15	
Dysmenorrhea					0.636
Yes	1,157	20.73	566	20.29	
No	4,424	79.27	2,224	79.71	
Medications during pregnancy					0.412
Yes	269	4.82	146	5.23	
No	5,312	95.18	2,644	94.77	
Pregnancy route					0.790
Conceived naturally	5,494	98.44	2,746	98.42	
IVF	83	1.49	43	1.54	
Ovarian stimulation	4	0.07	1	0.04	
Vaginal bleeding					0.380
Yes	511	9.16	272	9.75	
No	5,070	90.84	2,518	90.25	
Regular perinatal visits					0.109
Yes	4,545	81.44	2,312	82.87	
No	1,036	18.56	478	17.13	
Placenta previa					0.507
Yes	472	8.46	248	8.89	
No	5,109	91.54	2,542	91.11	
PIH					0.019
Yes	870	15.59	491	17.60	
No	4,711	84.41	2,299	82.40	
GDM					0.183
Yes	1,301	23.31	687	24.62	
No	4,280	76.69	2,103	75.38	
Pregnancy complicated by medical disease					0.596
Yes	1,283	22.99	627	22.47	
No	4,298	77.01	2,163	77.53	
Pregnancy complicated by surgical disease					0.719
Yes	96	1.72	45	1.61	
No	5,485	98.28	2,745	98.39	
Pregnancy complicated by gynecological disease					0.260
Yes	470	8.42	215	7.71	
No	5,111	91.58	2,575	92.29	
Time between last CS and pregnancy (months)^*^	73.23 ± 45.25		75.01 ± 47.42		0.211
Elected CS as last CS					0.438
Yes	702	12.58	345	12.37	
No	3,035	54.38	1,578	56.56	
Missing	1,844	33.04	867	31.08	
CS during labor as last CS					0.603
Yes	440	7.88	213	7.63	
No	1,991	35.67	1,011	36.24	
Missing	3,150	56.44	1,566	56.13	

*Values are presented as the mean ± SD and median (range). BMI: body mass index; PIH: pregnancy-induced hypertension syndrome; GDM: gestational diabetes mellitus; CS: cesarean section; IVF: in vitro fertilization*.

* *There were missing data in the variable, and 2,462 (44.13%) and 1,208 (43.30%) data were missing in the training set and validation set, respectively*.

### Risk Factors of PA

After examination and transformation of variables to fit the logistic regression model, variables were selected by the backward stepwise selection method (*P* < 0.05). The results of univariable analyses are shown in Table 2, and the multivariable logistic regression analyses are shown in Table 3. In the PA group, there were 57 (19.7%) patients with missing data in the time between last CS and pregnancy, 148 (51.0%) patients with elective CS as last CS, and 151 (52.1%) patients with CS during labor as the last CS. In the non-PA group, there were 2,406 (45.5%) with missing data in the time between last CS and pregnancy, 1,696 (32.1%) with elected CS as last CS, and 2,999 (56.7%) with CS during labor as last CS. In univariable analyses, in the presented data, the times between last CS, CS during labor as last CS and elective CS as last CS were analyzed, with the missing data overlooked. There was statistical significance only in the times between last CS and pregnancy; however, we excluded this in multivariable logistic regression due to the large percentage of missing data. The number of previous CS, number of vaginal bleeding, medications during pregnancy, and placenta previa were all found to be independent risk factors for PA (*P* = 0.044, *P* = 0.003, *P* = 0.003, and *P* < 0.001, respectively).

**Table 2:** Univariable analyses between the placenta accreta group and the non-placenta accreta group in the training set.

		**Placenta accreta (*n* = 290)**	**Non-placenta accreta (*n* = 5,291)**	***P*-value**
Age	<25	6 (2.1)	105 (2.0)	0.007
	25–30	59 (20.3)	851 (16.1)	
	30–35	97 (33.4)	2,296 (43.4)	
	35–40	101 (34.8)	1,713 (32.4)	
	≥40	27 (9.3)	326 (6.2)	
BMI before delivery		27.23 ± 3.59	27.79 ± 3.69	0.032
Gravity	1	1 (0.3)	124 (2.30)	<0.001
	2	73 (25.2)	2,170 (41.0)	
	3	80 (27.6)	1,498 (28.3)	
	4	79 (27.2)	937 (17.7)	
	≥5	57 (19.7)	562 (10.6)	
Parity	0	2 (0.7)	270 (5.1)	<0.001
	1	257 (88.6)	4,681 (88.5)	
	≥2	31 (10.7)	340 (6.4)	
History of vaginal delivery		9 (3.1)	105 (2.0)	0.190
Number of previous cesarean section	0	2 (0.7)	266 (5.0)	<0.001
	1	264 (91)	4,749 (89.8)	
	≥2	24 (8.3)	276 (5.2)	
Number of previous medical abortion	0	265 (91.4)	5,037 (95.2)	0.013
	1	17 (5.9)	182 (3.4)	
	≥2	8 (2.8)	72 (1.4)	
Number of previous artificial abortion	0	127 (43.8)	3,044 (57.5)	<0.001
	1	70 (24.1)	1,277 (24.1)	
	2	60 (20.7)	681 (12.9)	
	≥3	33 (11.4)	289 (5.5)	
History of Induction		7 (2.4)	141 (2.7)	0.796
Myomectomy		4 (1.4)	324 (6.1)	0.001
History of gynecological surgery		29 (10.0)	306 (5.8)	0.003
Regular menstruation		263 (90.7)	4,874 (92.1)	0.381
Menstrual flow	Hypomenorrhea	24 (8.3)	150 (2.8)	<0.001
	Normal	258 (89.0)	5,044 (95.3)	
	Menorrhagia	8 (2.8)	97 (1.8)	
Dysmenorrhea		76 (26.2)	1,081 (20.8)	0.018
Medications during pregnancy		30 (10.3)	239 (4.5)	<0.001
Pregnancy route	Conceived naturally	288 (99.3)	5,206 (98.4)	0.455
	IVF	2 (0.7)	81 (1.5)	
	Ovarian stimulation	0 (0.0)	4 (0.1)	
Number of previous vaginal bleeding	0	147 (50.7)	4,923 (93.0)	<0.001
	1	77 (26.6)	301 (5.7)	
	≥2	66 (22.8)	67 (1.3)	
Regular perinatal visits		232 (80.8)	4,313 (81.5)	0.518
Placenta previa		252 (86.9)	220 (4.2)	<0.001
PIH		12 (4.1)	858 (16.2)	<0.001
GDM		44 (15.2)	1,257 (15.8)	0.001
Pregnancy complicated by medical disease		48 (16.6)	1,235 (23.3)	0.007
Pregnancy complicated by surgical disease		3 (1.0)	93 (1.8)	0.488
Pregnancy complicated by gynecological disease		8 (2.8)	462 (8.7)	<0.001
Time between last CS and pregnancy^*^		91.14 ± 51.23	71.78 ± 44.43	<0.001
Elected CS as last CS^*^		109 (76.8)	2,926 (81.4)	0.166
CS during labor as last CS^*^		29 (20.9)	411 (17.9)	0.383

*Values are presented as the mean ± SD and number (percentage). BMI: body mass index; PIH: pregnancy-induced hypertension syndrome; GDM: gestational diabetes mellitus; CS: cesarean section; IVF: in vitro fertilization*.

* *There were missing data in the variable. In the placenta accreta group, there were 57 (19.7%) patients with missing data in the time between last CS and pregnancy, 148 (51.0%) patients with elective CS as last CS, and 151 (52.1%) patients with CS during labor as the last CS. In the non-placenta accreta group, there were 2,406 (45.5%) with missing data in the time between last CS and pregnancy, 1,696 (32.1%) with elected CS as last CS, and 2,999 (56.7%) with CS during labor as last CS*.

**Table 3:** Multivariable logistic regression analyses between the placenta accreta group and the non-placenta accreta group in the training set (*N* = 5,581).

	**OR**	**95% CI**	***P*-value**
Placenta previa			
Yes vs. no	119.30	80.33–177.18	<0.001
Medications during pregnancy			
Yes vs. no	2.57	1.37–4.83	0.003
Number of previous cesarean section			0.044
0	Ref.	Ref.	
1	3.56	0.76–16.76	
≥2	6.56	1.25–34.50	
Number of vaginal bleeding			0.003
0	Ref.	Ref.	
1	1.62	1.08–2.44	
≥2	2.11	1.30–2.41	

*OR, odds ratio; CI, confidence interval; Ref., reference*.

### Development and Validation of Nomogram

**Figure 1:**
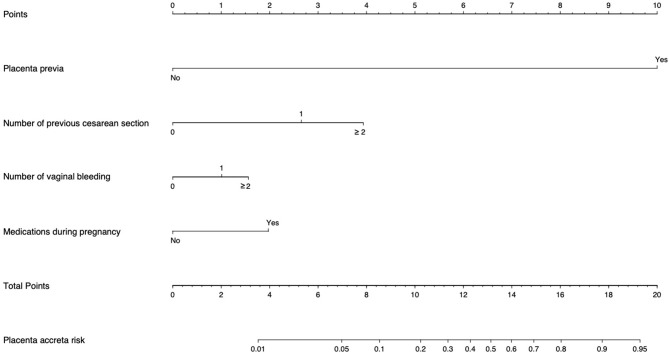
Placenta accreta Risk Assessment Tool. “Points” refers to point for the individual risk factor and add together to the “Total points.” “Placenta accreta risk” was calculated according to the ‘'Total points.” Example: For a patient with one previous CS (score = 2.6), with one vaginal bleeding (score = 1), with placenta previa (score = 10), and medication during pregnancy (score = 1.9), the total score is 15.5 corresponding to a 74% risk of placenta accreta (PA).

**Figure 2:**
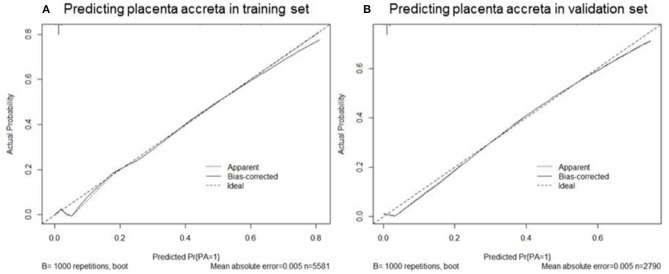
Calibration plots of nomogram to predict the probability of placenta accreta in the training set **(A)** and validation set **(B)**. The x-axis is the predicted placenta accreta calculated by the nomogram, and the y-axis is the observed placenta accreta. The “Ideal” is the ideal curve, and the solid line “Bias-corrected” is the actual curve.

**Figure 3:**
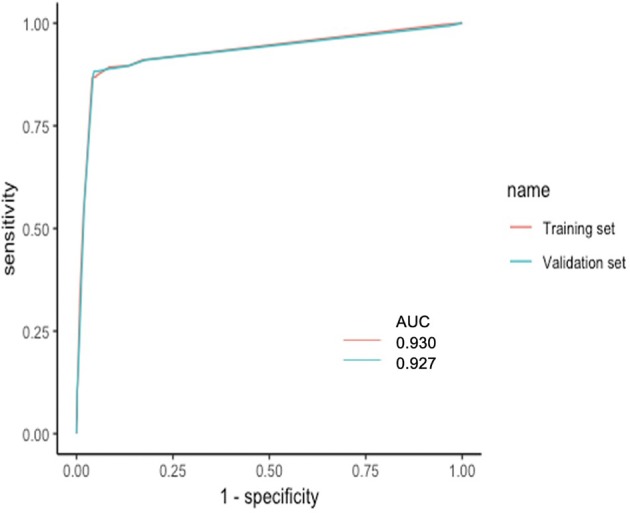
ROC curves of training set and validation set. The x-axis is the “1—Specificity,” and the y-axis is “Sensitivity”. AUCs were also presented with 0.930 and 0.927, respectively. AUC, area under the curve; ROC, receiver operating characteristic.

Based on the coefficients of the logistic regression model that identified independent risk factors of PA, a novel nomogram was then developed to predict the risk of PA (Figure 1). In the training set, Harrell's C-index was 0.93. Figure 2A shows the calibration plot of the nomogram in the training set. The x-axis is the predicted PA calculated by the nomogram, and the y-axis is the observed PA. Discrimination and calibration were found to be excellent in the validation set. The Harrell's C-index was 0.927. The nomogram was well-calibrated, with a good correlation between predicted and observed PA (Figure 2B). The receiver operating characteristic curves of training set and validation set are presented in Figure 3.

## Discussion

We developed and validated a new nomogram that included number of previous CS, number of vaginal bleeding, medications during pregnancy, and placenta previa as covariates for estimating the risk of PA in pregnancy with scarred uterus.

In our study, the incidence of PA was 5.2%, which was higher than previous studies. This can be explained by the fact that in this study, the study population was limited to scarred uterus patients who had a history of CS or myomectomy.

In our study, we found number of vaginal bleeding to be an independent risk factor for PA. Rac et al. thought that vaginal bleeding decreased with advancing gestation in pregnancies with PA (22). Furthermore, women with a non-adherent low-lying placenta or placenta previa had higher rates of bleeding when compared to PA (23). In other words, women with PA may not bleed as much as their non-adherent counterparts. However, we found that number of vaginal bleeding is an independent risk factor for PA. The difference may result from the inclusion of patients in our study, while the former study is associated only with patients with prior CS and persistent placenta previa. Placenta previa was thought to be the major risk factor for PA in a review (24). Of course, in our study, placenta previa is also an independent risk factor for PA and may be related to number of vaginal bleeding.

In this study, cases were included in the medication group regardless of the type of medication used during pregnancy. In previous studies, compared to non-users, medication users were more likely to have spontaneous abortion, preterm birth, stillbirth, neonatal death, children with congenital malformations, postpartum depression, and decreased birth weight (25–27). To date there is no literature on the association between medications during pregnancy and PA. In our study, we found that all medications used during pregnancy, regardless of the type of medication, may lead to PA. In univariable analyses, pregnancies complicated by disease, including gestational diabetes mellitus and pregnancy-induced hypertension syndrome, were associated with PA, but not independently. However, medication, which may be associated with various diseases during pregnancy, is an independent risk factor for PA. Medication may be taken during pregnancy not only to treat diseases during pregnancy but for other purposes as well; for example, some patients may use antibiotics to prevent infection, or take vitamins as nutritional supplement, or take them for protection of the fetus during pregnancy (steroid hormones and Chinese herbal medicines). Moreover, some patients with these diseases may not be treated with medication during pregnancy. Therefore, there is no established correlation between medication taken during pregnancy and diseases occurring during pregnancy; nevertheless, we speculate that medication taken for disease during pregnancy is an independent risk factor for PA.

In addition, there may be other factors at work that may explain why medication is an independent risk factor. PA is associated with decidual defects, abnormal trophoblast invasion, and abnormal placental angiogenesis (28). We doubt that medication in the fetal/placental circulation affects the secretion of certain factors in the placenta, which influence trophoblast invasion and angiogenesis of the placenta. Moreover, medications such as heparin and aspirin may affect the systemic blood circulation and the secretion of cytokines, which may lead to changes in maternal systemic and endometrial hormone levels, which may in turn affect endometrial decidualization, resulting in the implantation of the embryo and PA (29, 30). However, there is currently insufficient evidence to be certain whether medication during pregnancy is an independent risk for PA. Thus, from our study, we suggest that women with a scarred uterus, when using medications during pregnancy, should be aware of the risk of PA. Since decidualization and trophoblast invasion occurred between the first and early second trimesters (31), we assumed that medication taken after the early second trimester plays a minor role in PA. Furthermore, given that the route of administration of the medication, whether oral, vaginal, or intravenous, may result in different serum or endometrial medication levels, and also given that the pharmacokinetics of many drugs are altered during pregnancy (32), the doses of medication should also change during pregnancy. A different route and a changed dose could lead to a different degree of endometrial decidualization. In the future, the time, doses, and route of medication during pregnancy should also be studied in PA patients, in order to determine the association between PA and medication during pregnancy more clearly.

In our study, we found that a history of myomectomy is a risk factor for PA, but not an independent one, which is a similar finding to that of Gyamfi-Bannerman et al. (33). Previous studies have found an increasing risk of PA with an increasing number of cesarean deliveries (12, 13, 34). We also found a history of CS to be an independent risk factor. This finding may be explained by the potentially larger area of anatomical disruption from a CS scar as opposed to the smaller myomectomy scar.

Carusi (20) and Zeng et al. (35) both considered age to be a risk factor for PA. However, after stratification in multivariate regression, age was found not to be an independent risk factor for PA as a result of a scarred uterus in older patients.

Multidisciplinary teamwork and operator experience have been shown to reduce collateral damage, with several studies demonstrating that maternal morbidity is significantly reduced by delivery in a specialist center for PA patients (36–38). However, medical institutions at or below the county level cannot identify and diagnose PA at an early stage and lack experience in treating PA, due to the uneven distribution of medical resources and the uneven development of urban and rural areas in China, which leads in turn to poor maternal and perinatal outcomes. The nomogram of this study is based solely on clinical data (except for ultrasound images), and the validation using both the training set and the validation set revealed good discrimination and calibration. The nomogram can help doctors in primary hospitals to detect the risk of PA at an early stage in order to better manage a high-risk population so they may then refer patients to a specialist center to improve the maternal and perinatal outcomes.

The advantages of our study include the development of a novel nomogram to predict the risk of PA, which can be used in primary hospitals in northeast China, and even be extended to the whole of China. Furthermore, the cases in our study were taken from Shengjing Hospital affiliated to China Medical University, which is a tertiary hospital and the Obstetrical Critical Care Center in northeast China, and they are representative of the epidemiological trends evident in northeast China. Furthermore, our study was supported by the “The National Key Research and Development Program of Reproductive Health & Major Birth Defects Control and Prevention” project. However, in the future, larger prospective investigations involving multiple centers from the project, as well as larger numbers of patients, could be undertaken with longer periods of follow-up, to validate and improve the nomogram.

Despite these strengths, some limitations of the present study should be acknowledged. First, in spite of the large sample size of both the training set and validation set, an external validation would have strengthened our findings. Second, the retrospective nature of the study does not totally rule out that several important variables were not tested, such as the time between last CS and pregnancy, whether CS took place during labor, and whether the last CS was an elected CS. Also, the number of missing data was not negligible; hence, the “not available” variables were not introduced in the nomogram. Thus, the model including these variables needs to be developed and validated in the future. Third, the diagnosis of PA was a clinical one, which may result in inaccuracies in determining the actual degree of invasion; and the surgeon's experience could also affect the accuracy. Fourth, patients with non-scarred uterus were excluded, and such patients, following intrauterine gynecological surgery and artificial abortion, may have endometrial defects and PA. Therefore, the risk of PA in non-scarred uterus patients or multiple pregnancies should also be explored in the future.

Bowman et al. (39) suggested that when evaluated by a diverse group of ultrasound providers who are blinded as to the patient's clinical history, the diagnostic performance characteristics of ultrasound may not be as high as has been previously reported. Therefore, it is important to identify where there is a high-risk population and further improve the diagnostic accuracy rate of ultrasound. Therefore, we suggest that the nomogram is routinely applied to those patients with a scarred uterus to evaluate the risk of PA, especially in a primary hospital setting.

In conclusion, we developed and validated a nomogram for estimating the risk of PA in scarred uterus patients in China. Future validation of the present model and nomogram is warranted to confirm our findings.

## Data Availability Statement

The datasets generated for this study will not be made publicly available. All the clinic informations are generated from the HIS system of our hospital and followed up for a short time. It is patients' privacy and we should treat them confidentially.

## Ethics Statement

The study was approved by the Ethical Committee of Shengjing Hospital and China Medical University, and patients gave signed informed consent.

## Author Contributions

CQ and CL: concept. TY: development of the methodology and writing of the manuscript. NL: revision of the manuscript.

### Conflict of Interest

The authors declare that the research was conducted in the absence of any commercial or financial relationships that could be construed as a potential conflict of interest.
